# Alexithymia: Toward an Experimental, Processual Affective Science with Effective Interventions

**DOI:** 10.1146/annurev-psych-021424-030718

**Published:** 2024-12-03

**Authors:** Olivier Luminet, Kristy A. Nielson

**Affiliations:** 1 Research Institute for Psychological Sciences, UCLouvain, Louvain-la-Neuve, Belgium; 2 Fund for Scientific Research (FRS-FNRS), Brussels, Belgium; 3 Department of Psychology, Marquette University, Milwaukee, Wisconsin, USA; 4 Department of Neurology, Medical College of Wisconsin, Milwaukee, Wisconsin, USA

**Keywords:** affect, personality assessment, cognition, emotion regulation, psychosocial interventions, psychopathology

## Abstract

Alexithymia is a multi-dimensional personality trait involving difficulty identifying feelings, difficulty describing feelings, and an externally oriented thinking style. Poor fantasy life is debated as another facet. For over 50 years, the alexithymia literature has examined how alexithymia-related disturbances in perceiving and expressing feelings contribute to mental and physical disorders. We review the current understanding of alexithymia—including its definition, etiology, measurement, and vulnerabilities for both mental and physical illness—and its treatment. We emphasize the importance of further experimental and processual affective science research that (*a*) emphasizes facet-level analysis toward an understanding of the nuanced bases of alexithymia effects on neural, cognitive, and behavioral processes; (*b*) distinguishes between emotion deficits and emotion over-responding, including when over-responding is functional; and (*c*) clarifies when and how impairments occur for neutral and positively valenced information or contexts. Taken as a whole, a clarification of these issues will provide clear directions for effective and tailored alexithymia interventions.

## 1. INTRODUCTION AND HISTORICAL CONTEXT

Alexithymia is an important and fascinating domain of research and applications, as well as one that is still controversial. Alexithymia is fascinating because it shows that our psychological characteristics are related to our physiological, behavioral, and cognitive functions and even to our propensity to develop physical diseases. Alexithymia is thus at the center of the mind-body question that has been debated in the Western culture since the time of the ancient Greeks. Although today there is little doubt that our psyche can impact our soma, the manner in which this occurs is still a matter of scientific debate. For over 50 years, the alexithymia literature has actively contributed to this debate, considering whether the disturbances in perceiving and expressing feelings contribute to mental and physical disorders. Alexithymia is also still controversial because of persisting doubts stemming from its clinical and psychosomatic origins, issues of measurement validity, and the seeming inconsistency between some observations of excessive or even adaptive emotional expression and the emotional deficits shown by the majority of studies. Throughout this article, we address these issues together with a research agenda for the future in order to better understand the mechanisms of action and potential interventions that will help people with high alexithymia to better manage their personal life and their relationships. We also show that research on alexithymia is at the crossroads between health and social sciences, providing a rich mix of theoretical backgrounds, methodological approaches, and types of interventions.

Examining the roots of a construct helps us understand its developmental steps. This section highlights some key moments before the birth of alexithymia. The notion of alexithymia originated in Europe in psychosomatic medicine in the 1920s among doubts about body-mind dualism. Clinicians repeatedly reported that people with specific somatic diseases were unable to experience and identify their emotions as subjective feelings, had a lack of fantasies about feelings and instinctual drives, and were preoccupied with external events (e.g., Wittkower 1938). These researchers, fleeing Nazism in the 1930s, relocated to the United States, where psychosomatic medicine grew rapidly. This period was characterized by systematic observations examining emotional and cognitive processes in patients displaying one of the classic diseases thought to have a psychological etiology, such as peptic ulcer, asthma, and atopic dermatitis. Among these scholars, Alexander (1950) highlighted unconscious psychological conflicts, while Ruesch (1948) emphasized stunted development of personality and symbolic expression. MacLean (1949) developed a psychophysiological model tying psychosomatic illnesses to physiological stress resulting from deficits in verbal emotion expression. Others emphasized a lack of introspection (Horney 1952, Kelman 1952). In parallel, Marty & de M’Uzan (1963) developed the notion of *pensée opératoire*, a style of thinking that is utilitarian, pragmatic, and lacking fantasy. It is also characterized by seemingly ignoring emotional reactions during conflict or trauma and a lack of affective ties in relationships (McDougall 1982). Importantly, these theorists departed from a prevalent aspect of psychosomatic theory, that vulnerability to somatic illness is explained by an intrapsychic conflict, proposing instead an absence of processes (i.e., deficits) as responsible for increased vulnerability to disease. Taken together, their clinical observations described a pattern of reduced verbal and symbolic emotion expression, low imaginative ability, and a tendency to use physical action when stressed rather than mental processes.

In the 1970s, clinicians working with so-called psychosomatic patients advanced earlier theories to propose that psychological states could increase the vulnerability to physical diseases (Nemiah & Sifneos 1970, Nemiah et al. 1976). Their bottom-up approach, based on systematic clinical observations, marked the birth of the alexithymia construct. It provided an ideal template to improve our understanding of the reciprocal links between physical and mental health, focusing on how emotion-related psychological states contribute to the development of mental and physical illness. Yet, a valid, reliable assessment of alexithymia was elusive, which hampered empirical research into clarifying the definition and specific facets of alexithymia, the contexts in which alexithymia could predict dysfunctional emotion processing, and the emotional mechanisms of vulnerability to disease. Since the advent of valid alexithymia measurement in the 1990s, the field has grown at an exponential pace.

## 2. CURRENT UNDERSTANDING OF ALEXITHYMIA

To contextualize the last 50 years of research, we overview the definition and central facets of alexithymia together with its main etiologies, measurements, vulnerability factors, and construct stability. At each stage, we highlight where there is consensus versus theoretical, empirical, or clinical debate, suggesting ways to resolve these debates.

### 2.1. Definition

There is strong agreement that alexithymia involves at least three central facets: difficulty identifying feelings (DIF), difficulty describing feelings (DDF), and externally oriented thinking (EOT) (Luminet et al. 2018, 2021b; Preece & Gross 2023; Taylor & Bagby 2021). DIF highlights deficits in recognizing and distinguishing between emotional states. In stressful situations, for example, people with higher DIF have difficulty knowing whether they feel anger, fear, or anxiety, instead primarily experiencing undifferentiated distress and discomfort. DIF also includes difficulty distinguishing one’s feelings from internal bodily sensations and states, known as interoception (Desmedt et al. 2023). Related to DIF, and yet a distinct facet, DDF is characterized by deficits in describing feelings to others. It is tied directly to the etymology of the term, which literally means “having a lack of words for feelings” (i.e., *a*, a lack of; *lexis*, language; and *thymos*, mood or emotion).

EOT, first described as pensée opératoire (Marty & de M’Uzan 1963), was proposed as a paucity or an absence of fantasies related to drives and feelings, which then restricts thought. It thereby produces a cognitive style dominated by preoccupation with external details (e.g., the weather) at the expense of internal events and responses (Nemiah et al. 1976). Since its inception, however, the role of fantasies has been debated. Poor fantasy (PF) life refers to a lack of daydreaming, imaginal activities, and abstract/symbolic aspects of life. The current EOT facet focuses on the external, operative thinking style, with debate surrounding the contribution of PF. Some consider PF as a central aspect of alexithymia that is difficult to measure because it is influenced by social desirability (Taylor & Bagby 2021; see Section 2.3). Others exclude it from the construct because the results are sometimes the opposite of predictions (i.e., greater rather than less fantasizing in alexithymia) and because fantasizing does not factor into the same construct with DIF, DDF, and EOT (Preece et al. 2017; see also Preece & Gross 2023 and Section 3.1).

One unfortunate outcome of the label “alexithymia” is that it is often misunderstood as only a language deficit. Importantly, alexithymia is not categorical, wherein someone either has or does not have it. Rather, it is a multidimensional personality trait, meaning that it is a stable mode of thinking, reacting, and behaving with a variety of dimensions that are present to some degree in all persons. Indeed, two identical alexithymia scores can represent meaningfully different patterns of facet characteristics. Furthermore, detailed analyses comparing alexithymia to the five-factor model of personality domains (Costa & McCrae 1992) demonstrate that alexithymia is a unique construct (see the sidebar titled Alexithymia and Its Unique Association with the Five-Factor Model of Personality) reflecting individual differences in emotional experience, cognition, and behaviors, which do not correspond to any single personality domain or lower-order trait (Luminet et al. 1999). Finally, while the early literature characterized high alexithymia as an inability to understand and process emotions, today there is clear consensus that alexithymia facets vary in intensity along a continuum that represents difficulty rather than inability. This change also acknowledges that interventions can effectively reduce alexithymia scores (see Section 4).

### 2.2. Etiology

The field of alexithymia gathers scholars from many domains of the medical and psychological sciences. They share views on what alexithymia is but not necessarily on how to explain it. Indeed, the mechanisms leading to alexithymia are not definitively known. In addition to its psychodynamic etiological beginnings (see Section 1), there are multiple, potentially overlapping pathways, including behavioral, cognitive, physiological, and social mechanisms (see Luminet et al. 2018, Lumley et al. 1996). Indeed, both health and disease are influenced by each of these factors (Porcelli & Taylor 2018). Here we address some of the major hypotheses that explain the etiology of alexithymia from three primary, and sometimes overlapping, perspectives: development, cognition, and emotion regulation. Although more models exist, these perspectives provide ideal foundations for empirical studies investigating the psychological processes of alexithymia and for designing intervention studies based on those processes.

#### 2.2.1. Developmental models.

A fundamental approach to the etiology of alexithymia focuses on the progressive appearance of alexithymia during development. This developmental process is usually attributed to an environment in which the parents/caregivers fail to bond with children (Li et al. 2023a) or fail to encourage the understanding and sharing of emotions (Lane & Schwartz 1987). Thus, children miss crucial steps in emotional development, such as needed feedback through facial expressions, postures, or intonation; learning the meaning of emotional words and the context in which they are used; and how, when, and where to share emotional aspects of their lives. This limited context and exposure further impair an understanding of the meaning of physiological responses to emotion (e.g., arousal) and how to translate them into feelings and words. Specifically, this model contends that a person’s levels of emotional awareness and responding depend on how well emotion knowledge or schemas have developed. Indeed, Lane et al. (2015) described alexithymia as affective agnosia, whereby there is a deficit in the ability to make meaningful mental representations of emotion that also impacts the expression and communication of emotion. Relatedly, alexithymia can develop in childhood or in adulthood, acquired after physical trauma (Hobson et al. 2019, Hogeveen & Grafman 2021) or psychological trauma as in Krystal’s (1995) attribution of high alexithymia prevalence in Holocaust survivors to their early traumatic experiences.

Attachment is a construct that represents the closeness of connections among humans, which are initially formed between infants and caregivers. Childhood trauma, abuse, or neglect can lead to developing an insecure attachment style, where the person becomes detached from (avoidant attachment) or excessively preoccupied with (anxious attachment) feelings and relationships, which contributes to affect dysregulation and maladaptive affect coping strategies (see Schimmenti & Caretti 2018) and poor emotion schema development (Bucci 1997b; see Panayiotou et al. 2018). Importantly, childhood trauma and insecure attachment styles are empirically linked to alexithymia in adulthood through DIF and DDF, with the avoidant style most predictive of alexithymia (see Schimmenti & Caretti 2018).

#### 2.2.2. Cognitive models.

Cognitive models underline the role of various aspects of information processing in alexithymia. These models emphasize the translation of emotion states to cognitive states, which are thought to occur in two stages. The first one is the subsymbolic stage (Bucci 1997a,b), where the early and fleeting result of an experience triggers adaptive physiological and behavioral responses, including relatively undifferentiated subjective appraisals such as intensity, valence (i.e., positive or negative tone), novelty, importance, and familiarity. This has recently been characterized as the early, automatic sensory processing stage (Morie et al. 2022). Second, the symbolic (Bucci 1997a,b) or cognitive (Morie et al. 2022) stage includes both internal processing, or thoughts connected to prior experiences and responses, and external processing, or the communication of the event and the initial impressions through language. Thus, language is the symbolic cognitive function that (*a*) facilitates making sense of one’s current emotive context and sensations, (*b*) links knowledge and experience for labeling these experiences as feelings, and (*c*) communicates these feelings to others (Lindquist 2017). The progression from subsymbolic to symbolic processing allows language to also provide tools to gain distance from and reflect about initial emotive experiences.

Early milestones in language development importantly assist with acquiring knowledge and concepts; along with social experiences with caregivers, this knowledge prepares children for attending to and using social cues both behaviorally and linguistically (see Hobson et al. 2019, Lee et al. 2022). The language hypothesis of alexithymia (Hobson et al. 2019, Lee et al. 2022) proposes that alexithymia develops due to (*a*) weak emotion concept formation, resulting in DIF; and (*b*) poor social-linguistic development that impacts the ability to access and apply emotion words, resulting in DDF. Indeed, empirical research shows a consistent deficit across most domains of language functioning in alexithymia, at least in emotive contexts (e.g., 15 of 16 studies; Luminet et al. 2021a), including reduced perception of nuances among emotion concepts (emotional granularity; Lee et al. 2022) and reduced social appropriateness (pragmatics; Jakobson & Pearson 2021). Thus, limited or deficient transitions from subsymbolic- to symbolic-level processes in alexithymia keep the person connected to the initial event and its intense bodily sensations, hampering the linking of concrete experiences with images and words that allows one to interpret and communicate the subjective emotional experience (Taylor & Bagby 2004).

Cues, which vary by context, are important to determine the meaning of emotional stimuli and situations (e.g., tears of either joy or sadness; Morie et al. 2022). Limited symbolic processing in alexithymia produces both difficulty distinguishing among emotions and difficulty distinguishing among contextual cues (Diaz & Prinz 2023). Moreover, the external style in alexithymia may lead to an emphasis on describing contextual details, such as the surrounding scene, rather than to using emotion words to describe the event (e.g., Luminet et al. 2004). Interpretative biases are notable in alexithymia, particularly about bodily sensations. Being able to detect and make meaning of bodily signals (interoception) informs us about potential dangers. Sudden sensations such as heat or cold, difficulty breathing, or stomach or throat tension are typically triggers that, if ignored, result in prolonged exposure to or feelings of unresolved threat. Yet, continuous bodily monitoring would mask distinctions between threatening and benign situations, hampering appropriate responses to genuine threat. Interoceptive dysfunction contributes to disrupted symbolic representations of feeling states and, thus, to a difficulty communicating them and relying on other (i.e., nonsymbolic) processes to effectively identify and regulate emotion (Morie et al. 2022). Interestingly, although the relationship between alexithymia and interoception is mainly negative, the magnitude and direction of correlations vary across studies (Zamariola et al. 2018), partly due to which alexithymia facets are examined. However, fundamental concerns about validation of interoception assessments have cast doubt on drawing strong conclusions about interoception at this time (e.g., Desmedt et al. 2023).

#### 2.2.3. Emotion regulation models.

In order to achieve or reinstate homeostasis, people have an extraordinary capacity to adapt the meaning they attribute to disruptions caused by emotive situations. Emotion regulation, the process of assessing, controlling, and modifying emotional responses to meet goals and social expectations, operates consciously and unconsciously at the physiological, behavioral (expressive), and cognitive (experiential) level (Luminet et al. 2021a). The attention-appraisal model (Preece et al. 2017), based on dysfunctional emotion regulation in alexithymia, was founded on psychometric factor analysis, impaired emotion schema development (Lane & Schwartz 1987), and Gross’s (2015) extended emotion regulation process model. Four sequential stages are theorized: situation occurrence, attention to the stimulus, identification and making sense of the stimulus (i.e., appraisal), and response (where emotion regulation occurs). This deficit model (see Section 3.2.1) proposes that difficulty attending to stimuli (attributable to EOT) and difficulty appraising stimuli (due to DIF and DDF) cause external and internal distraction, which results in maladaptive emotion regulation strategies aimed at disengaging from negative emotions. Empirical findings consistently show poorer emotion regulation and excessive use of maladaptive strategies in high alexithymia, which are linked to negative mental and physical health outcomes and poorer interpersonal functioning (Luminet & Zamariola 2018, Preece et al. 2023). However, it is notable that individual differences and context can also have adaptive influences on emotion regulation even in alexithymia, wherein either adaptive or maladaptive strategies are employed depending on the situation. This model does not yet adequately account for adaptive responding (see Section 3.2.1).

The attention-appraisal model represents at least a subtle shift (Preece & Gross 2023) of emphasis away from the original pensée opératoire concept (Nemiah & Sifneos 1970) by focusing on avoidance of emotion and deficits in attention and by excluding imaginal activity from the construct (Taylor et al. 2023). Supporting the model, recent studies show greater rather than reduced imaginal activity in alexithymia and poor correspondence between fantasy and the alexithymia construct (Preece & Gross 2023, Preece et al. 2023). Furthermore, as empirical evidence supports alexithymia-related deficits across the continuum of automatic to controlled attention (Luminet et al. 2021a, Vermeulen et al. 2018), and studies examining the facets of alexithymia most often link EOT to these deficits (Correro et al. 2021, Wiebe et al. 2017), empirical attention data support the attention-appraisal model. Thus, it does not appear that tension between the original view of pensée opératoire and the role of EOT in the attention-appraisal model is necessary. However, the model’s predictions about DIF and DDF in appraisals are not yet clear. DDF and EOT, but not DIF, have empirically supported roles in appraisals (Luminet et al. 2021a), although valence is seldom included, preventing a full appreciation of the relationship between the facets of alexithymia and appraisals (see Section 3.1.3).

### 2.3. Measurement

Alexithymia research was not able to substantively progress until the advent of the first psychometrically valid instrument, the self-reported Toronto Alexithymia Scale (TAS; Taylor et al. 1985). TAS was soon revised as the 20-item TAS-20 (Bagby et al. 1994a,b), which is now the most used instrument worldwide (Bagby et al. 2020). Joining TAS-20 are two newer self-report instruments, the Bermond-Vorst Alexithymia Questionnaire (BVAQ; Bermond et al. 2015) and the Perth Alexithymia Questionnaire (PAQ; Preece et al. 2018). Given the difficulty of self-identifying emotional states in alexithymia, we also briefly compare TAS-20 with two clinician-observer scales, the Toronto Structured Interview for Alexithymia (TSIA; Bagby et al. 2005) and the TAS-20 Informant Form (TAS-20-IF; Bagby et al. 2021).

#### 2.3.1. Twenty-item Toronto Alexithymia Scale.

The original self-report TAS (Taylor et al. 1985) included 26 items over four dimensions (DIF, DDF, EOT, and PF). However, as PF was influenced by social desirability, it was removed and the TAS-20 (Bagby et al. 1994a,b) was proposed to sufficiently capture both PF and pensée opératoire (Marty & de M’Uzan 1963) within the EOT facet. Over 30 years, TAS-20 has demonstrated excellent psychometrics, including convergent and divergent validity, and a stable three-factor structure across many languages in student, community, and patient populations (Bagby et al. 2020). Internal consistency is good for total, DIF, and DDF scores. Lower internal consistency for EOT (often below 0.70) has been attributed to response bias, due to a high proportion of reverse-scored items and the inclusion of two potentially unique constructs, low valuation of emotional life and pragmatic thinking style (Bagby et al. 2020). A recent meta-analysis reinforced the validity of the three-factor structure and also emphasized that facet scores provide a more accurate picture of alexithymia than a general score (Schroeders et al. 2022). Indeed, two identical TAS-20 total scores can represent wholly different facet patterns, just as with the five-factor model personality domains (Costa & McCrae 1992). Thus, while a total score might suffice for clinical diagnosis, empirical results strongly support evaluating the moderating impact of TAS-20 facets when investigating emotion and information processing (see Section 3.1.2). Furthermore, Schroeders et al. (2022) found good measurement invariance between clinical and nonclinical samples but not across translations, suggesting that TAS-20 clinical validity may be limited to Western contexts or that alexithymia might manifest differently across cultures. Future research should address cultural influences on alexithymia and its measurement and also consider TAS-20 facets alongside close constructs such as mentalizing, empathy, and emotion regulation to better place it into a larger nomological network.

#### 2.3.2. Bermond-Vorst Alexithymia Questionnaire.

The self-report BVAQ (40 items, in two parallel 20-item forms) includes five dimensions: DIF, DDF, EOT (all identical to TAS-20), an independent PF dimension, and “emotionalizing,” which evaluates the degree to which emotional events evoke arousal (Bermond et al. 2015). Emotionalizing is controversial and is considered a correlate of alexithymia rather than a central facet (Luminet et al. 2004, Taylor et al. 2000). Regardless, BVAQ provides two separate, higher-order dimensions: cognitive alexithymia (DIF, DDF, EOT), which overlaps with TAS-20 total (*r* = 0.80; Vorst & Bermond 2001), and affective alexithymia (PF, emotionalizing), which is only weakly correlated with TAS-20. Ongoing debates include whether BVAQ explains alexithymia better than another measure—incremental validity—and whether five dimensions rather than three are warranted.

#### 2.3.3. Perth Alexithymia Questionnaire.

The 24-item self-report PAQ (Preece et al. 2018) mirrors the TAS-20 facets (DIF, DDF, EOT) but has a different emphasis than TAS-20, based on the attention-appraisal model (see Section 2.2). Thus, the PAQ EOT facet contains a single dimension, failure to focus attention on emotions, with items often tapping avoidance strategies (e.g., “I tend to ignore how I feel”). PAQ also captures valence, separating positive and negative emotions in DIF and DDF with separate facets (i.e., negative valence denoted as N-DIF and N-DDF, and positive valence denoted as P-DIF and P-DDF). PAQ has high concurrent validity with TAS-20 where content is comparable (total *r* = 0.67, DIF *r* = 0.57, DDF *r* = 0.59; Zahid et al. 2024) and also for EOT, despite differing conceptualizations and content (*r* = 0.46), suggesting they measure similar constructs. PAQ internal consistency is high, often above 0.90, which may be clinically desirable (Preece et al. 2018) but also might suggest some redundant items (Streiner 2003). The stronger EOT internal consistency of PAQ compared to TAS-20 might be due to its more focused EOT conceptualization and lack of reverse-scored items.

The incremental validity of PAQ over TAS-20 was recently studied against need for cognition, psychological mindedness, and subjective interoception (Zahid et al. 2024), with the results raising doubts related to the need for cognition and interoception. Yet, the findings can also be debated as the study compared PAQ facets only with the TAS-20 total, excluded relevant criterion variables (e.g., emotion regulation, attention, appraisals; Luminet et al. 2021b), and used a limited self-report interoception instrument (e.g., Vlemincx et al. 2023).

#### 2.3.4. Clinician-observer evaluation versus self-report.

Considering the possible validity issues of asking someone with alexithymia to self-report on emotion processing, a comparison with clinician and observer assessments is valuable. The Toronto Structured Interview for Alexithymia (TSIA) (Bagby et al. 2005), a 24-item clinician-administered interview, includes four facets (DIF, DDF, and EOT, all from TAS-20, and PF from TAS), as well as two higher-order dimensions, affect awareness (DIF, DDF) and operative thinking (PF, EOT). Although recent network analyses cast further doubt about PF as a central alexithymia facet (Watters et al. 2016), TSIA and TAS-20 total scores have high concurrent validity (Bagby et al. 2005), reinforcing self-report as comparably valid with a clinician interview but faster to administer. Similarly, observer ratings such as with the newer TAS-20-IF (Bagby et al. 2021) may offer contrasting or complementary assessments.

### 2.4. Alexithymia as a Vulnerability Factor

Numerous correlational studies empirically support that alexithymia is associated with various somatic and psychological outcomes and physical and mental disorders (Porcelli & Taylor 2018). These studies do not, however, address cause–effect relationships. That is, they cannot distinguish whether alexithymia is a vulnerability factor influencing the onset or course of these disorders or is merely a state reaction to the presence of a disorder or accompanying distress. To determine vulnerability, the relative stability of alexithymia must be established against acute stress, or alexithymia must be assessed longitudinally with somatic outcomes.

#### 2.4.1. Relative stability in the presence of acute stress.

Alexithymia was long considered a state-dependent phenomenon, due to its positive correlations with depression and neuroticism (Honkalampi et al. 2000). Although some studies alternatively supported a trait view of alexithymia, showing stability across time (Salminen et al. 1994), most only evaluated absolute stability (i.e., the extent to which alexithymia scores change over time) rather than relative stability (i.e., the extent to which relative differences among individuals remain the same over time). Relative stability, in the context of acute changes in stress or symptoms, is required to demonstrate that alexithymia is a vulnerability factor for somatic or psychological disorders. Relative stability is estimated using measures of covariation, including test-retest correlations, hierarchical regressions predicting follow-up scores using baseline scores with stress factors (e.g., depression, anxiety) as covariates, and changes in scores predicted by stress factors to verify that stress factors explain only trivial amounts of alexithymia change.

Although there are exceptions, the preponderance of evidence, particularly when TAS-20 is used, supports the relative stability of alexithymia in clinical and nonclinical samples. For example, outpatients treated pharmacologically for depression had an absolute reduction of both depression and alexithymia, but depression change did not explain alexithymia change (Luminet et al. 2001). Similarly, in breast cancer, changes from pre-surgery to six-month follow-up showed that alexithymia change was not substantively explained by situational factors (Luminet et al. 2007). A comparable outcome was obtained with alcohol withdrawal inpatients, despite the extreme changes in psychological distress and physiological stress that occur during treatment (de Timary et al. 2008). Interestingly, DDF and EOT evidenced absolute stability in this study, although DIF reduction modestly correlated with depression, suggesting it may be partially related to mood variations. In nonclinical university students, an increase in psychological distress across the academic term, as the exam period drew near, did not explain alexithymia change (Mikolajczak & Luminet 2006). Additionally, 11-year general population studies that considered many relevant covariates also demonstrated relative stability (Tolmunen et al. 2011) along with generally higher scores in older age (Hiirola et al. 2017). Overall, alexithymia studies show low absolute stability but high relative stability, affirming it as a vulnerability factor for mental and physical disorders.

#### 2.4.2. Associations with somatic illness.

Voluminous evidence in small and large, mostly cross-sectional, studies supports an association between alexithymia scores and somatic illness (Porcelli & Taylor 2018). For instance, in >1,000 participants and controlling for many covariates, TAS-20 total was significantly associated with hypertension and carotid atherosclerosis, corresponding to 10% of atherosclerosis plaques in those with higher alexithymia (Grabe et al. 2010). Importantly, longitudinal studies also show the association of alexithymia to illness, which is needed to establish causal links. For example, large population-based studies have shown, after controlling for various contributing factors, that alexithymia doubles or triples 5-year mortality risk (Kauhanen et al. 1996) and significantly increases cardiovascular disease-related death (Tolmunen et al. 2011). Alexithymia also significantly predicted 3- to 12-month pain outcomes after breast cancer surgery, after controlling for other relevant factors (Baudic et al. 2016). While seemingly conclusive, these studies represent a small longitudinal literature in alexithymia that must grow. Longitudinal designs contextualized within alexithymia have the potential to radically improve our understanding of emotion processing as a nonhomogeneous function with complex and varying physiological, affective, cognitive, and trait-related contributions (Luminet et al. 2021b). However, few studies have simultaneously considered multiple aspects of this interface; longitudinal designs are the ideal scenario for future research tackling this need.

#### 2.4.3. COVID-19 and alexithymia.

The COVID-19 pandemic, an international public health crisis characterized by vast numbers of hospitalizations and deaths, caused widespread panic, isolation, and amplification of mental health disorders (Wang et al. 2020). Other health-related behaviors, such as alcohol consumption and eating, have also been investigated as outcomes of the pandemic. Each of these factors is highly relevant to alexithymia. Thus, pandemic-related studies can importantly inform our understanding of the facets and impacts of alexithymia over periods of collective public stress. We highlight two studies as illustrations. Li et al. (2023b) used latent profile analysis to examine alcohol use among young adult parents over a 10-month period during the COVID-19 pandemic. Considering depression, anxiety, and alcohol use, they identified distinct patterns of alcohol use and transitions that alexithymia facets differentially predicted. Specifically, DIF was associated with risky drinking at baseline, increased distress-related non-risky drinking, and ongoing risky drinking. Importantly, EOT uniquely predicted both sustained risky drinking and the development of risky drinking during the pandemic. EOT was suggested to lead to escalating risky drinking through deficient executive functioning (EF) (see Correro et al. 2021) and stress-related avoidant coping (Wiebe et al. 2017). Similarly, McAtamney et al. (2021) reported that emotional eating, the tendency to (over)eat in response to negative emotions, was associated with both DIF and DDF during the pandemic, with the effect operating indirectly through emotion dysregulation. Overall, these results highlight the specific roles of alexithymia facets in increasing health vulnerabilities, which may be particularly heightened by major public health crises. Moreover, they emphasize the importance of including alexithymia assessment in health evaluations and treatment planning, because alexithymia is a crucial risk factor for poorer outcomes, particularly in those with greater stress and emotional symptoms and low resilience (e.g., poor coping).

## 3. FUTURE DIRECTIONS IN ALEXITHYMIA RESEARCH

In this section, we address central goals for future alexithymia research, with a primary focus on nonclinical populations (see Section 4 for clinical interventions). First, we discuss construct conceptualization, including a clarification of the core alexithymia dimensions, using a facet-oriented approach and measurement refinements, as well as the integration of alexithymia research with affective and neurocognitive science. Second, we elaborate a vision for adopting an experimental and processual approach in future research, highlighting some themes as important emphases to achieve that vision.

### 3.1. Conceptualization

Although an extensive alexithymia literature has evolved over the past 30 years, greatly facilitated by the development of the TAS-20 instrument (see Section 2), a number of ongoing issues about the conceptualization of alexithymia either are unresolved or have been further amplified by the development of newer instruments and progress in experimental behavioral and neurocognitive research. Here we discuss themes about conceptualization that must be addressed to facilitate progress in alexithymia research and practice.

#### 3.1.1. Core dimensions: the role of fantasizing.

While there is general agreement over 30 years of research with the TAS-20 that the core DIF, DDF, and EOT facets are central dimensions of alexithymia, there are ongoing debates regarding the contribution of PF and, relatedly, the approach to EOT, which differs across instruments (see Section 2). The suggestion that EOT captures fantasizing without a separate dimension (Taylor & Bagby 2021) is unsatisfactory, particularly in light of the clear three-factor structure of TAS-20 (Schroeders et al. 2022) and the questionable consistency of PF with the other alexithymia dimensions (Preece & Gross 2023). The weak internal consistency of EOT in TAS-20 (Bagby et al. 2020), especially compared with EOT in PAQ (Preece et al. 2018), also suggests that EOT needs further refinement. We suggest that broad adoption of independent, parallel measures of PF would help to finally resolve the role of fantasizing in alexithymia and assist in refining alexithymia measures, including the EOT dimension.

#### 3.1.2. Facet-oriented approach.

A majority of TAS-20 variance is reflective of a single construct, suggesting that total scores are sufficient for diagnostic cutoffs (Bagby et al. 2020). However, facet-level analysis is superior in research, particularly since different instruments consider a different content and number of facets (Schroeders et al. 2022). A psychometric analysis can furthermore discern unique information from facets that the total score cannot provide (e.g., bifactor modeling; Reise 2012). Indeed, clinical profile analyses can reveal, from the same total score, vastly different facet patterns to justify tailoring of intervention approaches. From a research perspective, facet analysis is recommended, facilitating a processual approach to connect specific characteristics with specific maladaptive and adaptive responses at the various stages of processing (see Section 3.2.1). Indeed, the potential interactions among facets and different processing stages remain nearly unexplored (but see Correro et al. 2021). Further exploration could importantly inform our understanding of alexithymia impacts on somatic and mental health.

#### 3.1.3. Measurement refinements.

There are still multiple refinements to consider in alexithymia measurement. First, although DIF and DDF are defined identically and correlate highly across self-report instruments, we recently found they differed when using TAS-20 versus PAQ (N-DIF, N-DDF) (E. Grimm, E. Ledoux & O. Luminet, unpublished manuscript). Thus, there may be subtle but important DIF and DDF instrument differences yet to be explored.

Second, and relatedly, does assessing by valence, as used by PAQ, add incremental validity to the alexithymia construct? While clinically derived instruments (TAS-20, BVAQ) do not distinguish valence, the PAQ considers the efficient management of both negative and positive emotions to be important to healthy mental and physical life (Preece & Gross 2023). This approach also has empirical support, for example, from the negative associations between alexithymia and anhedonia (Pinna et al. 2020) and the positive emotions facet in the five-factor model of personality (Luminet et al. 1999). Preece et al. (2024) further reported that both N-DIF and P-DIF predicted anxiety and depression, suggesting that the identification of both negative and positive emotions contributes to psychopathology. Indeed, latent profile analyses also distinguished valence-related high-psychopathology clusters separately within negative (N-DIF, N-DDF) and positive (P-DIF, P-DDF) emotions, with no profile combining positive and negative valence across the same facet. However, the valence specificity of these correlational findings needs complementary positive and negative emotion induction studies to demonstrate that the correspondence between N-DIF and negative affect is stronger than the correspondence between N-DIF and positive affect, and vice versa for P-DIF. Studies evaluating whether valenced items in TAS-20 add nuance and accuracy to outcome measures could further illuminate the role of valence.

Third, measurement would be greatly enhanced by complementary performance-based approaches, using cognitive and behavioral abilities to model the socio-emotional processes that underlie each facet (Robinson et al. 2019). For instance, standardized measures that require extensive emotional expression in response to specific prompts about emotional situations can objectively assess DDF. Indeed, the Levels of Emotional Awareness Scale (LEAS; Lane et al. 1990) does this for a somewhat different purpose. A textual analysis of LEAS responses, rather than using its traditional scoring, or a similar type of task could provide a performance-based assessment of DDF. Indeed, McEnaney & Ryan (2024) recently reported a similar objective approach, using computer-scored responses from the little-used Alexithymia Provoked Response Questionnaire (Krystal et al. 1986). Similar tasks that require emotion identification would tap DIF, and eye-tracking tasks (e.g., dwell time) could evaluate EOT (Wiebe et al. 2017). Routine inclusion of such measures would provide empirical determination of the central alexithymia facets and assist with the honing of survey measures. Separate performance-based measures of PF and EOT would also help clarify the functional differences between these constructs.

Fourth, an important priority is for greater inclusivity in alexithymia measurement, including by age, education, culture, sex, and gender. Only limited study of these factors exists, with mixed findings that lack causal relationships. Research has not yet addressed gender, but sex differences consistently suggest that alexithymia is greater in men, tied to EOT and DDF, which may result from men being socialized to avoid expressing emotion (Ryder et al. 2018). Alexithymia is also higher when education and socioeconomic status are low (Honkalampi et al. 2000). Age effects are less consistent, possibly due to birth cohort differences (Hiirola et al. 2017). Finally, despite ample evidence that culture shapes emotion processing, the main proxy of culture in alexithymia is language (Ryder et al. 2018). Yet, undergraduate studies show higher alexithymia in people with East Asian origins compared to North American samples (e.g., Lo 2014), primarily via EOT (see Ryder et al. 2018). As EOT validity has been questioned in non-Western cultures (Schroeders et al. 2022), it is unclear whether EOT assessment requires cultural adaptation or whether differences are attributable to cultural adherence rather than pathology (Ryder et al. 2021).

#### 3.1.4. Integration with affective and neurocognitive sciences.

Originating in psychosomatic medicine, alexithymia research lacks a full integration, conceptually or methodologically, with affective or neurocognitive sciences. Although progress has been made (Luminet et al. 2021a,b), we highlight here several overarching themes that can assist with the integration of these fields and further progress.

##### 3.1.4.1. Affective approaches.

Alexithymia restricts the cognitive and affective resources that allow people to temper distressing emotions and impulsive actions (Luminet et al. 2021a). These are key aspects of affective science. Prior to experiencing feelings, automatic appraisals are activated by emotive events, such as determining whether an event is (un)pleasant or goal-(ir)relevant. Emotive events also invoke a state of action readiness, such as preparation to flee a frightening situation (Scherer & Moors 2019). These key steps are dissociated in alexithymia, with reduced appraisals [e.g., lower (un)pleasantness] and increased action tendencies (e.g., proneness to impulsive, aggressive responses; Luminet et al. 2021a), which can possibly be explained by maladaptive emotion regulation (Edwards & Wupperman 2017). Experimental research intentionally integrating appraisals and action tendencies can assist in better revealing their specific roles in behavior and cognition.

Importantly, as some situations are more difficult to appraise and act upon than others, multiple contextual factors are important to consider in research, including experiential avoidance (EA) (Panayiotou et al. 2015); modality (i.e., facial, vocal, bodily reactions), which can influence the categorization and labeling of feelings (Scherer & Moors 2019); and task or situational complexity, which can impair performance in high alexithymia (e.g., Ihme et al. 2014a,b). Additionally, even if no differences are found in primary emotion responses (e.g., to neutral, happy, sad videos), there may be secondary emotional effects (e.g., anger after a funny film; Karlsson et al. 2008) suggesting that measurement of a full range of concomitant affect is important. Similarly, emotional context and content are not synonymous, and each can have moderating influences that should be distinguished. Specifically, the content of the material experienced (e.g., affective meaning of words), the environmental state in which it is experienced (e.g., music induction), and the person’s physiological state (e.g., arousal) can each influence appraisal, action, and the resulting feelings and cognition (e.g., Nielson & Lorber 2009, Nielson & Meltzer 2009, Nielson et al. 2005). For example, emotional material often impairs memory in alexithymia (Luminet et al. 2021a), but it can enhance memory when it is particularly salient (Meltzer & Nielson 2010) or when it is congruent with the context, such as joy words accompanied by happy music or anger words accompanied by angry music (Vermeulen et al. 2010).

Another contribution from affective science that could benefit alexithymia research is the distinction between affect intensity, the magnitude of emotions when confronted with highly relevant appraisals (e.g., high novelty, unexpectedness, and importance), and affect frequency, which is the number of similar affective occurrences (Diener et al. 1985). While frequent positive affective experiences are considered healthy, high positive intensity is related to psychological dysfunction, such as bipolar disorder (Diener et al. 1985). Alexithymia has been linked to hypomania, with high-DIF men having reduced positive affect frequency with greater positive affect intensity (Fantini-Hauwel et al. 2015). Together, these findings suggest a compounding of vulnerability to mental illness in alexithymia.

##### 3.1.4.2. Neurocognitive approaches.

Emotion influences attention across the cognitive continuum from early automatic orienting to later controlled processing, with competitive biases prioritizing salient or meaningful inputs (Yiend 2010). Saliency thus prioritizes stimuli such as danger signals and preferred information, with downstream cognitive effects resulting in reduced or enhanced processing, depending on motivations and goals. Alexithymia research consistently reveals deficits in early attention and later controlled attention for emotive stimuli and threat-related biases (Luminet et al. 2021a, Vermeulen et al. 2018), such as greater attention to and maintenance of (i.e., over-responding to) illness words (Lundh & Simonsson-Sarnecki 2002, Meltzer & Nielson 2010). These biases hint at a generalized deficit in cognitive control, or EF, which includes various higher-order cognitive processes (e.g., inhibition, task shifting, updating memory) that underlie controlled action and goal-directed behaviors (Miyake et al. 2000). Emotional salience modulates EF to refocus attention to situation-relevant responding, which is crucial for effective emotion regulation. Moreover, accumulating evidence demonstrates a generalized deficit of cognitive control associated with DIF that is not limited to emotion (Correro et al. 2021, Henry et al. 2006, Koven & Thomas 2010). As impaired cognitive control results in more stimulus-driven processing with downstream cognitive consequences, EF could account for early attention deficits (EOT), salience-related biases in attention and memory (DIF) (Correro et al. 2021, Henry et al. 2006, Koven & Thomas 2010; see Luminet et al. 2021a), and subsequent maladaptive emotion regulation (Preece & Gross 2023). Thus, additional systematic experiments that integrate cognitive and affective approaches, with intentional interrogation of the temporal progression of processing, can aid a cohesive understanding of alexithymia.

Corroborating cognitive evidence, there is extensive evidence of alexithymia-related alterations of the brain networks underlying emotion, interoception, and self-perception, namely, the salience and executive networks (e.g., Goerlich & Aleman 2018). The salience network, which includes the amygdala, anterior cingulate, insula, and prefrontal cortex (PFC), integrates internal and external sensory and emotive cues, helping to monitor conflict and prioritize novel, meaningful, and potentially rewarding stimuli (Menon & D’Esposito 2022). The executive network overlaps anatomically with the salience network but is anchored in the PFC. It links prioritized information with decision-making and action, including functions such as maintaining and manipulating information in current memory and cognitive control over goal-directed behavior (Menon & D’Esposito 2022). Alexithymia, particularly DIF, is associated with aberrations in the functions and connectivity within both networks (Hogeveen & Grafman 2021), and neurophysiological studies show these are early disruptions that result in downstream conscious processing deficits (Goerlich 2018, Luminet et al. 2021a). While neurofunctional alexithymia studies typically employ emotional stimuli or tasks, we recently showed DIF-related frontal cortex aberrations also during non-emotive tasks (Otteman et al. 2023, Polking et al. 2023). Our findings extend evidence of smaller brain volumes and altered connectivity at rest [i.e., in the default mode network (DMN)] when no task or stimulus is present (e.g., Goerlich & Aleman 2018). The DMN overlaps extensively with the semantic network, which is also relevant to alexithymia as it manages storage and access to knowledge and concepts (Binder & Desai 2011). EF fundamentally influences all these processes. Thus, taken with generalized alexithymia-related deficits in autonomic (see Panayiotou et al. 2021) and hormonal (e.g., Goerlich & Votinov 2022) functioning, there is increasing appreciation that alexithymia may have a broad neural basis that transcends emotion processing. Further integration of affective and neurocognitive approaches, with attention to include neutral stimuli and contexts, will allow a fuller understanding of the foundations of alexithymia (see Section 3.2.2).

### 3.2. Envisioning an Experimental and Processual Approach

**Figure 1:**
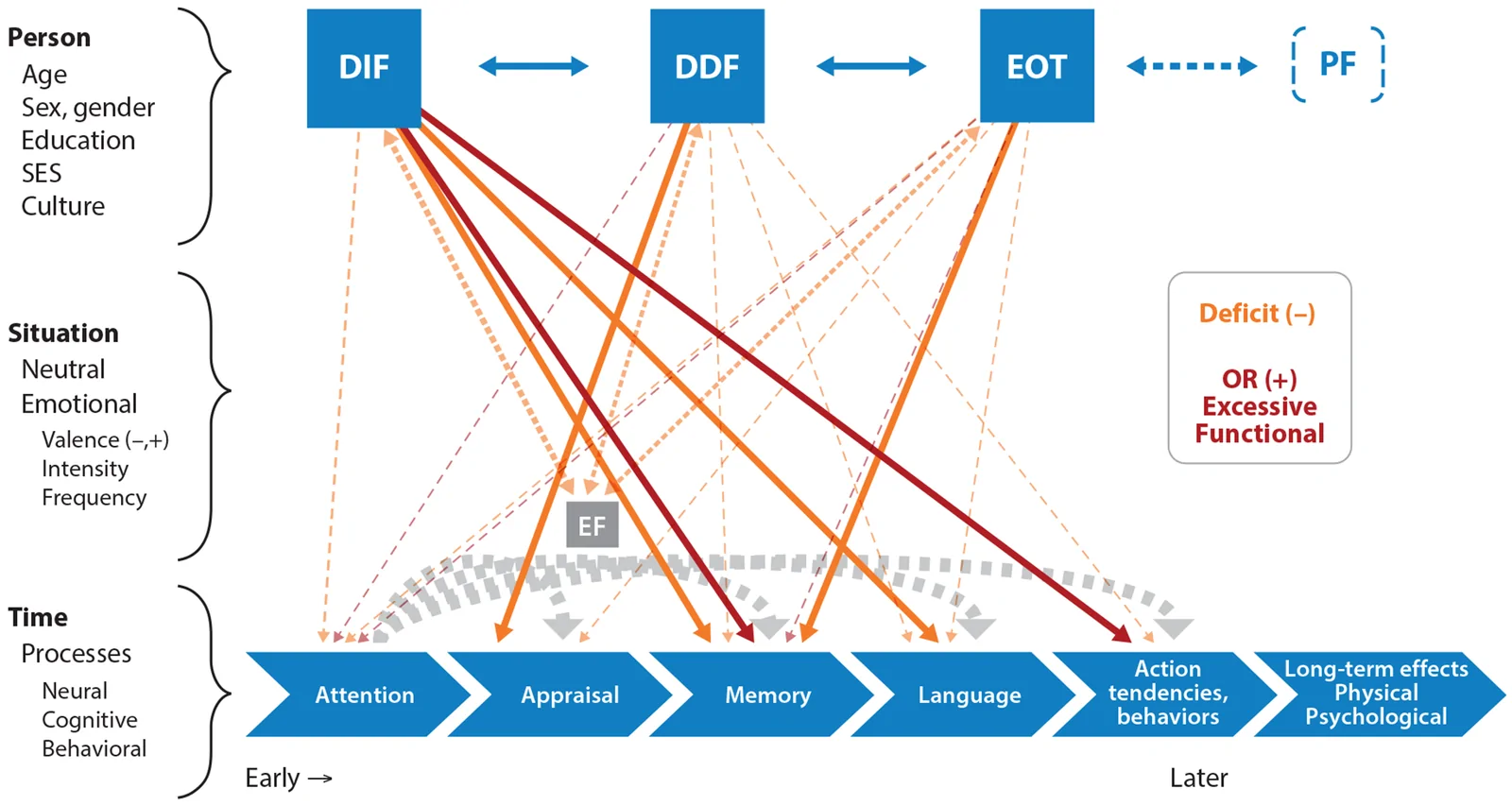
Overview of the complex interplay of alexithymia facets—difficulty identifying feelings (DIF), difficulty describing feelings (DDF), and externally oriented thinking (EOT) on the cognition–emotion interface. Poor fantasy (PF) life is debated as a core facet but requires further study. Alexithymia facets can be influenced at the person level by individual differences, while situational factors from neutral to emotional (negative and positive) influence the manner in which the facets alter neurocognitive and behavioral processes. Early processes, including attention and appraisal, have downstream effects on later processes including memory, language, action tendencies, and behaviors. The influences of alexithymia facets include deficits (i.e., impaired processes in emotional and/or neutral contexts) and over-responding (OR), which includes excessive responding and functional (i.e., beneficial) responding. The facets also interact with executive functioning (EF) to influence each of the processes (*dotted arrows*). The small number of existing studies investigating these processes have shown complex facet effects; the consistency and number of findings showing deficits or OR are shown by the intensity of the links (solid/bold to dashed/opaque; see Luminet et al. 2021a). These influences and interactions are thought to lead to the long-term physical and psychological outcomes associated with alexithymia, thereby suggesting specific routes for interventions. Experimental studies are needed that examine these intricate relationships and outcomes at multiple levels of person, situation, and process. Abbreviation: SES, socioeconomic status.

The existing empirical literature on cognitive and emotional processes in alexithymia is correlational. A shift to experimental designs and a systematic, processual approach will allow researchers to draw causal inferences and systematically study where in the processing stream alexithymia facets moderate functioning (Luminet et al. 2021a,b). This would adopt an interactionist framework, considering individual differences in emotional responses as concomitant effects of the person, the situation, and the time (Kuppens & Verduyn 2017). Therefore, an important goal is to specify the conditions under which alexithymia facets modulate emotional and cognitive responses, thereby following a process-oriented personality psychology approach (e.g., Quirin et al. 2020, Robinson et al. 2019) and utilizing more objectively assessed processes such as attention, perception, appraisals, cognitive control, and behaviors (Robinson et al. 2019). Here we present first steps and priorities for this vision, which will be aided by increased transdisciplinary collaborations across multiple sites (see Figure 1).

#### 3.2.1. Distinguishing deficits from over-responding and functional responding.

Since its inception, the literature has characterized alexithymia as dysfunctional responding and deficits in emotion identification and communication and in cognitive processing and emotion regulation (see Taylor et al. 2000), such as poor attention to and interest in emotional stimuli. Thus, the field characterizes alexithymia as a vulnerability factor for dysfunction due to disorganized, undifferentiated, and poorly integrated emotional schemas that cause an inability to focus on and accurately interpret information about emotions (Luminet et al. 2021b). Although typically appropriate, this vulnerability approach has biased the literature to consider only deficits. This ignores two conditions: instances of over-responding and of advantageous, functional responding.

Over-responding occurs early, such as with an intense emotional reaction (e.g., crying outbursts, impulsive aggression; Edwards & Wupperman 2017) that is detectable by observers, with subsequent avoidance strategies (e.g., suppression; Preece et al. 2023) that mask the response from observers and even from the person experiencing them. Thus, over-responding might be frequent in alexithymia but is not obvious, leaving it neglected as a research theme. Preliminarily, EOT has been related to mostly deficits, while DIF is frequently linked to over-responding, and memory studies typically show deficits, while behavior studies find over-responding (Luminet et al. 2021a,b) (see Figure 1). Similarly, functional responding is typically overlooked. For example, although less attention is directed to emotional information (i.e., a deficit), this can be functional by reducing intrusive rumination (Luminet et al. 2004) and by decreasing engagement with distracting or threatening stimuli (Vermeulen et al. 2006). Indeed, reduced background distraction can even allow enhanced memory in alexithymia, including when congruent background music is played with memoranda (Vermeulen et al. 2010) or when neutral words are presented with (less-attended) emotive words (Dressaire et al. 2015, Meltzer & Nielson 2010). Like over-responding, functional responding can be followed by maladaptive strategies. Importantly, the distinction between deficits and over-responding is a key process-related difference in alexithymia. A full understanding of this distinction, preferably through systematic, multi-process studies (see Section 3.2.6), will illuminate the processing timeline and influences that could be tapped for interventions, just as a better understanding of functional responding could be leveraged to target appropriate processes in treatment (see Section 4), thereby also bolstering self-efficacy and confidence and, therefore, treatment efficacy (e.g., Luminet et al. 1999).

#### 3.2.2. Non-emotional contexts and material.

Many systematic alexithymia studies have reported contrasting findings about impaired processing of emotional material, with unimpaired processing of nonemotional information. Typically, these studies included emotional and nonemotional material in the same task. Yet, recent studies have highlighted alexithymia differences even when only neutral information is presented, with evidence of both deficits and over-responding depending on the context (Luminet et al. 2021a). Memory studies provide a good example. When neutral and emotional materials are both included, high-DIF participants exhibit enhanced immediate memory for neutral stimuli (Dressaire et al. 2015, Ridout et al. 2021), while at least five studies reported EOT-related deficits for immediate and long-term retention of neutral memoranda, particularly when only neutral items are presented (Luminet et al. 2021a) (see Figure 1). Additionally, nonemotive EF tasks consistently show poorer performance in alexithymia, including for inhibitory control, task switching, and perseverative errors (see Vermeulen et al. 2018). These deficits are linked to DDF for processing speed and to DIF for fluency (Vermeulen et al. 2018) or even for general EF when using a composite EF score (Correro et al. 2021). Similarly, DIF has been implicated in aberrant frontal cortex activation during nonemotive EF tasks (Otteman et al. 2023, Polking et al. 2023). Moreover, poorer EF can explain poor memory, but only in those with high EOT (Correro et al. 2021). Furthermore, neutral material effects might be moderated by task difficulty or salience. For example, when combined with emotional material, neutral material gains advantage, particularly when EOT is high, because emotional material receives less priority due to its lower salience in alexithymia. Therefore, more resources can be allocated to neutral information, resulting in better memory depending on the context (Dressaire et al. 2015, Luminet et al. 2021a). Similarly, DIF and EOT have potentially opposite or interacting effects, depending on the context.

#### 3.2.3. Interpersonal and collective processes.

While process-oriented studies mainly examine the impact of alexithymia on individual outcomes, more studies are now examining cognition–emotion interactions beyond the intraindividual level through interpersonal outcomes (Luminet et al. 2021b). Overall, they suggest alexithymia is characterized by impaired processing of others’ emotional states, deficits in empathy, and compromised prosociality (Grynberg et al. 2018). Where facets were examined, higher DIF was associated with poorer memory for anger- and happiness-related social interactions (Ridout et al. 2021) and slower decision making about others’ communications ( Jakobson & Pearson 2021). Higher DDF was associated with poorer understanding of others’ emotional communications (Ridout et al. 2021). During gaming with either humans or bots, and when unaware of which type of partner, people with higher EOT had poorer ability to reach consensus with human players (who sometimes act irrationally) but not with the always-rational bots (Gvirts & Dery 2021).

#### 3.2.4. Long-term effects.

Empirical alexithymia studies rarely consider long-term follow-up or persistence of changes in effects over time. Perhaps most emblematic are memory studies: Of those, 70% measured retention within one minute after encoding, while only a few have assessed retention up to 24 hours later (Luminet et al. 2021a). However, memory consolidation for lasting memory evolves over time, and deficits (or enhancements) can be either compensated or exacerbated over time. For instance, one study found poorer immediate memory for neutral words in alexithymia, but a post-encoding arousal manipulation effectively enhanced memory consolidation in both low and high alexithymia, leaving no alexithymia deficit after 24 hours (Nielson & Meltzer 2009). Similarly, mixed stimuli recalled 45 minutes after semantic encoding were impaired in alexithymia for negative words but enhanced (i.e., over-responding) for illness words and neutral words (Meltzer & Nielson 2010). Thus, alexithymia effects can differ by context and over time.

#### 3.2.5. Multi-process, multi-method approach.

To date, alexithymia studies have almost exclusively examined only one aspect of emotion or information processing. Instead, taking a multi-process approach would definitively explain the processes affected by alexithymia and how they are temporally, functionally, and conceptually related. Indeed, alexithymia influences both conscious, deliberate (explicit) processing and early, automatic (implicit) processing (e.g., Goerlich 2018, Luminet et al. 2021a, Vermeulen et al. 2018), which suggests that early implicit processes cause downstream effects on conscious processing. Systematic studies could tap this processing continuum, clarifying these relationships and informing both construct theory and therapeutic targets. Dissociations are also better informed by a multi-process approach. Luminet et al. (2004) found alexithymia deficits in emotion labeling, which could have been attributed to deficits in appraisal, attention, memory, or avoidance tendencies. Fortunately, a multi-process approach helped clarify that high-alexithymia participants talked and thought about emotional events, but they gave less focus to the meaning of them and had excessive physiological reactions to emotion. Indeed, many studies have shown dissociations of cognitive-affective processes in alexithymia, such as deficits in appraisals, language, and memory but over-responding in actions (Luminet et al. 2021a,b). Furthermore, the few studies that simultaneously considered physiological, behavioral, and cognitive components showed positive intercorrelations in low alexithymia, as is often found in normative samples, but dissociations in high alexithymia, where some components were activated while others were de- or in-activated (see Panayiotou et al. 2018). Coupling of components may function to increase emotional awareness (Luminet et al. 2021b), while their dissociation may provide a process explanation for both emotion awareness deficits (as in the three-process model; Smith et al. 2018) and ubiquitous negative mental and physical health outcomes in alexithymia (e.g., Morie & Ridout 2018). Together, these findings illuminate the intersecting and situationally specific relationships among emotional components and emphasize the value of multi-component studies for a fuller understanding of the interface between cognition and emotion generally, and of individual and personality differences specifically.

Related to the advantages of a multi-process approach, research designs employing a multi-methods approach are advantageous. They allow researchers to test outcome consistency across several measures of the same process and offer opportunities to more robustly measure a process by combining like measures into composites. Multi-method approaches to alexithymia assessment can specifically enrich our understanding of the alexithymia construct as well as improve its assessment. For instance, the relationships between alexithymia and emotional facial expression labeling differ when using self-report (TAS-20) or observer-report (TSIA) (Ihme et al. 2014a,b). The addition of performance-based assessments tapping each facet would further clarify process, outcome, and measurement distinctions. Indeed, whether considering assessment measures or outcomes, multi-methods provide a stronger case for construct validity (Hoemann et al. 2021).

#### 3.2.6. Open science in alexithymia.

It is notable that psychometric studies in alexithymia originate from three primary but separate networks of researchers in Toronto (TAS-20), Amsterdam (BVAQ), and Perth (PAQ). Moreover, despite the number of international researchers who study alexithymia, there is little large-scale collaboration. This siloed approach raises broader issues surrounding the importance of advancing open science for future progress in the study of alexithymia. Open science refers to multiple practices surrounding transparency, accessibility, and reproducibility in research. This movement includes overall agreement about the need for greater attention to research replication, as well as acknowledgment of the uncertainties surrounding best practices and barriers to replication. Crüwell et al. (2019) provide a very helpful overview of studies that have addressed these issues, specifically highlighting the need for transparency in reporting detailed empirical, analytical, and statistical methods; the accessibility of data and publications (i.e., open access); the intentional engagement of confirmatory research practices (with preregistration); and a commitment to direct and conceptual (extended) replication studies.

Greater, deliberate commitment to open science in the field of alexithymia has multiple advantages. In addition to improving research quality, accessibility, and reproducibility, it would specifically assist with dismantling silos and encourage multi-site collaboration and more multi-experiment, larger-sample studies, which would have significant advantages for theory advancement (e.g., Correro et al. 2021). Moreover, it would better encourage and allow for combining data sets to protect from or validate findings against low statistical power. For example, Vermeulen et al. (2006) used three experiments to examine affective priming, showing that alexithymia scores moderated the effect of angry, but not sad or happy, primes. A further analysis across the samples was then conducted to validate the finding against the possibility that nonsignificant effects were due to low statistical power. The specificity of the effect on anger primes was robustly reaffirmed with this larger data set, even across multiple negative affect scales. This finding opens the possibility of further extending comparisons with data from other laboratories and paradigms, such as from neuroimaging, where similar anger effects have been shown (e.g., Kano et al. 2003). Thus, collaborative studies across broader networks of investigators, using unified protocols, and conforming to an open science framework will substantively advance developments in alexithymia measurement and theory.

## 4. SOCIETAL IMPACT AND NEED FOR TREATMENT

Alexithymia prevalence is estimated at 7–13% in the general population, with much higher rates (e.g., 30%) in clinical populations, especially in eating disorders and autism (e.g., 50%) (see Grynberg et al. 2018, Morie & Ridout 2018, Porcelli & Taylor 2018, Schimmenti & Caretti 2018). It is a transdiagnostic risk factor for physical and mental health disorders (Porcelli & Taylor 2018) and dysfunctional interpersonal relationships (Grynberg et al. 2018; see Section 2.4). Some alexithymia risks are more distal, such as high prevalence in women who smoke during pregnancy (40%; Linn et al. 2020) and in violent crime offenders with antisocial profiles (Strickland et al. 2017). Indeed, high-alexithymia adolescents exhibit reduced altruism and greater victim mindset, hostility, and indifference toward others, which are key risk factors for developing chronic antisocial behaviors (Pepe et al. 2023). Thus, the influences of alexithymia far transcend the individual, posing vast economic and societal consequences that require a large-scale commitment to intervention. Given its roots in early childhood (see Section 1), prevention should be primary as a deliberate target in parenting courses and early childhood education. Beyond prevention, early identification and intervention are crucial for reducing the health and well-being impacts of alexithymia on the individual and the economic burden it poses to society. But is treatment effective for alexithymia? Here we overview our current understanding of psychological treatment approaches in alexithymia.

### 4.1. Psychosocial Interventions

Psychotherapy, such as cognitive behavioral therapy (CBT), guides people in exploring and understanding their emotions with the goal of improving their interactions in life and relationships. It requires intensive self-reflection that presumes skills that are impaired in alexithymia: an awareness of emotion states and the ability to appraise, discuss, and act on them. People with high alexithymia are frequently aloof or emotionally distant in relationships, including with a therapist, which can influence the therapist’s attitude toward the patient; strong countertransference has been noted since the original descriptions of the construct (e.g., Nemiah et al. 1976). Indeed, systematic reviews indicate that patients with high alexithymia have poorer psychotherapy outcomes in >50% of studies, particularly linked to DIF (see Ogrodniczuk et al. 2018, Pinna et al. 2020. Although alexithymia does not typically prevent therapeutic gains nor lead to worsening of the treated condition, these findings clearly show the importance of considering alexithymia in treatment planning.

Despite poorer outcomes for people with alexithymia in treatment for other conditions, a crucial question is whether psychotherapy can effectively reduce alexithymia. The literature is small, underpowered, heterogenous, and often poorly controlled, with alexithymia investigated only as a secondary outcome. However, systematic reviews show that alexithymia can be significantly reduced when alexithymia-specific skills are targeted (e.g., Cameron et al. 2014, Ogrodniczuk et al. 2018). Specifically, when CBT is multi-modal, including psychoeducation related to emotion processing, there is greater efficacy. For example, studies that included training in awareness of internal experiences, bodily sensations, emotion awareness and expression, or emotion regulation demonstrated significantly reduced alexithymia in clinical and nonclinical samples (e.g., Burger et al. 2016), reduced interpersonal problems (DIF; Ogrodniczuk et al. 2012), and showed maintenance for up to 24 months (Beresnevaite 2000). Generally, protocols including structured activities, skill building, and homework assignments (Leweke et al. 2009) or computerized activities (Morie et al. 2015) were most effective, perhaps by promoting problem-focused coping that leverages EOT in the person’s favor (Quilty et al. 2017).

Although group therapy adds social demands that might be challenging for people with alexithymia, others might prefer it as less demanding or less threatening than individual treatment (Leweke et al. 2009), and it might afford better skill learning through observation, interaction, and feedback from peers (Ogrodniczuk et al. 2011). Dialectical behavior therapy (DBT) is a highly structured evolution of CBT that emphasizes psychoeducation in emotion regulation skills and mindfulness, usually in both individual and group sessions. The skills typically targeted by DBT are relevant to each of the three core facets of alexithymia (DIF, DDF, EOT). A systematic review of DBT studies in mental health populations found significant reduction of alexithymia (primarily DIF) in six of eight studies (75%; Salles et al. 2023) and maintenance for as long as 24 months (Reilly et al. 2022). Mindfulness teaches objective, distanced observation of thoughts, sensations, and emotions, altering one’s relationship to experience by engaging the PFC in EF and emotion regulation strategies (Lyvers et al. 2014). Importantly, a meta-analysis of four randomized controlled trials of mindfulness in nonclinical samples demonstrated a significant reduction of alexithymia with >3 months of training (Norman et al. 2019). An app-based training in both mindfulness and socio-emotional dyadic treatment also significantly reduced alexithymia (DIF, DDF) and increased interoceptive awareness, although dyadic treatment had greater effects than mindfulness (Silveira et al. 2023). Further, EA, the tendency to avoid aversive thoughts, sensations, and situations, is linked to DIF (e.g., Panayiotou et al. 2015, 2021) and can explain much of the relationship between distress (e.g., depression, anxiety) and alexithymia (Torunsky et al. 2023). Although not yet evaluated in alexithymia, even a single session of DBT has been shown to reduce EA and maintain the benefit for three months (Bernstein et al. 2021).

With the roots of the alexithymia construct in psychodynamic theory, alexithymia treatment using psychodynamic therapy has been extensively debated (e.g., Quilty et al. 2017). Psychodynamic therapy is typically a lengthy process that immerses the individual in self-reflection about how past experiences, particularly from childhood, are related to their current mindset. It relies more heavily on insight, verbal skills, and self-reflection than CBT or DBT. Thus, debates center on its suitability to alexithymia relative to other approaches. Indeed, some studies have found efficacy with psychoeducation but not with psychodynamic therapy in alexithymia, while others have shown promise when such components are used within a multi-modal approach (see Ogrodniczuk et al. 2011, Pinna et al. 2020).

### 4.2. Cognitive Approaches

EF deficits emerge when cognitive control processes are unable to maintain priority of goal-oriented behavior over automatic processing. Deficits of EF not limited to emotive contexts have recently been reported in alexithymia (Correro et al. 2021), fitting with early through late (controlled) attention deficits (Luminet et al. 2021a) and altered neural activity and connectivity in frontal salience and executive networks (Goerlich & Aleman 2018; see Section 3.1.4.2). Various approaches exist to target the modulation of perception and attention, such as hypnosis, exposure therapy, and progressive muscle relaxation, in which a therapist guides the person to alter their phenomenological experiences. For example, in panic disorder, treatment including progressive muscle relaxation and exposure therapy improved DIF and DDF and sustained it for 6 months independent of panic outcomes (Rufer et al. 2010). Additionally, a nonpatient, high-alexithymia university sample given 8 weeks of emotion-focused mental imagery hypnosis treatment had significantly reduced TAS-20 total scores (Gay et al. 2008). As part of a multi-modal intervention, these approaches could assist in managing and reducing the impact of sensory experiences, allowing better skill building. Independently, these approaches can more indirectly modulate sensory and attentional processes than CBT/DBT, perhaps being better tolerated as less threatening than traditional therapy (Gay et al. 2008).

Direct training of EF has been used in contexts such as brain injury and aging. Although not evaluated for alexithymia, goal management training (GMT; Levine et al. 2011) is a type of training that might have potential for alexithymia. GMT is a manualized, group-based training with approximately 20 hours of psychoeducation, mindfulness, and homework focused on increasing awareness and self-monitoring of EF. GMT teaches the individual to monitor performance; to occasionally interrupt ongoing, automated behaviors; and to bring primary goals to the forefront, managing them by creating subgoals (Levine et al. 2011). In both clinical and nonclinical populations, on its own or combined with psychotherapy, meta-analyses have shown significant and sustained effects of GMT on various EF tests (except speed of processing), everyday activities, long-term memory, observer EF ratings, and patient mental health ratings (Stamenova & Levine 2019). The focus on EF may provide an additive treatment to psychotherapy to enhance skill building in mindfulness, or as a standalone approach that could more indirectly improve the cognitive functions impacted by alexithymia.

### 4.3. Alternative Approaches

The primary limitation to cognitive training of any kind, as with any psychotherapy approach, is meta-cognition—the insight, self-awareness, and interoception needed to assess and moderate behavior. Indirect or implicit approaches to skill training could be effective alternatives or adjuvant interventions in alexithymia. Neurofeedback uses electroencephalography with operant conditioning to train individuals to up- or down-regulate specific neural activity during video game–like activities (Omejc et al. 2019). Although these approaches suffer from nonstandard protocols, uncertain mechanisms, and varying efficacy, they are used broadly in clinical and healthy populations (Omejc et al. 2019). Indeed, in healthy adults, a single 30-minute neurofeedback game where subjects attempted to alter a rocket’s speed, which was controlled by infralow-frequency (<0.1 Hz) brain activity, led to increased connectivity in the salience, language, and visual networks (Dobrushina et al. 2020). In subclinical alexithymia, a follow-up pilot study (*N* = 9) used 15 neurofeedback sessions with a game that only required participants to mentally travel an artificial landscape whose speed and brightness were controlled by infralow-frequency electroencephalography. This showed increased connectivity in the salience, language, and visual networks and significantly decreased total TAS-20 score (Dobrushina et al. 2022). These results suggest that neurofeedback has the potential to improve emotion and social functioning.

Another indirect, nonpharmacological intervention, neurostimulation [e.g., high-definition transcranial direct current stimulation (HD-tDCS)] enhances functional brain states and connectivity, including reductions in psychiatric symptoms (e.g., Frohlich & Townsend 2021). Ample evidence of altered neural functioning in alexithymia in the salience and executive networks (see Section 3.1.4.2) suggests that frontal neurostimulation could be an effective intervention. Indeed, HD-tDCS in the frontal cortex enhances attention-related eye tracking and emotion regulation (Subramaniam et al. 2023). In alexithymia, college students given repeated right inferior-frontal HD-tDCS exhibited increased attention to happy and neutral (not sad) face stimuli (Zhang et al. 2022), suggesting that HD-tDCS improved functioning in the salience and executive networks.

Although exercise benefits psychological health (Smith & Merwin 2021) and is neuroprotective (Won et al. 2021), exercise interventions are understudied in alexithymia. Yoga, for example, is mindfulness oriented, and initial studies show it can reduce depression, anxiety, and alexithymia (Jonsson et al. 2020). Intensive aerobic exercise can also significantly reduce pain and alexithymia (Torlak et al. 2022). Thus, exercise might be an important complement to multi-modal treatment.

### 4.4. Future Treatment Directions

To better understand the efficacy and value of treatment for alexithymia, randomized controlled trials are needed comparing different treatment approaches with the full range of behavioral, cognitive, emotional, interpersonal, and neural outcome assessments; long-range follow-up is also needed to determine the stability of effects. Notably, pharmacotherapy was not directly addressed herein, as such treatments have targeted comorbid conditions (e.g., depression). However, stress- and sex-related hormones have been suggested as possible targets of intervention (Goerlich & Votinov 2022; but see Mierop et al. 2020), which might have value as part of multi-modal psychotherapy.

## 5. CONCLUSIONS

Alexithymia, a multi-dimensional personality trait at the crossroads between health and social sciences, has developmental, cognitive, and emotion regulation roots. Herein we addressed the important contexts in which alexithymia can be detrimental for physical and mental health, with the aim of facilitating further construct refinements and the development of effective, targeted treatments. To achieve this goal, we highlighted the occasions in which over-responding to emotion can be adaptive and the crucial distinctions that alexithymia facet-level analysis provide toward disentangling the conditions and contexts that lead to deficits or over-responding. Similarly, although the foundations of alexithymia lie in psychosomatic diseases that emphasize deficits and dysregulation specifically for negative emotions, we also highlighted that alexithymia is characterized by reduced positive emotion experience and disturbances in processing neutral information and during neutral contexts. Corroborating neural findings further suggest that alexithymia reflects more generalized dysfunction not limited to emotion processing. Thus, we urge future research to pursue a deliberate experimental, processual approach that distinguishes early from later processing; integrates affective and neurocognitive sciences; includes a facet-level analysis with multiple processes, measures, tasks, and valences; and assesses long-term outcomes. This agenda is ideal for further assessment and construct refinement as well as for developing targeted, effective interventions.

## ALEXITHYMIA AND ITS UNIQUE ASSOCIATION WITH THE FIVE-FACTOR MODEL OF PERSONALITY

Alexithymia was established and sustained as a construct by comparing it to existing, well-established personality structures, where it consistently demonstrated uniqueness. That is, TAS-20 correlated well enough with other constructs to be meaningful, but not so much that it was redundant (Bagby et al. 1994b). For example, facets within the domains of the five-factor model of personality (Costa & McCRae 1992) are also associated with emotions (e.g., Neuroticism with negative and Extraversion with positive emotions), and when correlated with TAS-20, a unique and complex pattern emerged. Associations were positive with Neuroticism (overall), negative with Extraversion (e.g., low experience of positive emotions, low assertiveness), negative with Openness (e.g., low value of emotion, low interest in new activities), and negative with Agreeableness (e.g., low altruism) (e.g., Luminet et al. 1999), which is linked also to low empathy (e.g., Grynberg et al. 2018). Thus, alexithymia is not related simply to decreased affect but rather to a dissociation between elevated distress and a reduced experience of positive affect (Luminet et al. 2021a). Moreover, alexithymia scores reflect individual differences not characterized by any single dimension or facet within the five-factor model but rather by a complex admixture of narrow personality traits (Luminet et al. 1999).

## SUMMARY POINTS

1. Alexithymia can be assessed validly with self-report measures, which can be complemented by other-report measures (in severe alexithymia). Objective performance measures are sought to add further validity and construct refinements.
2. Alexithymia reflects various disturbances in the processing of information, from the early stages (e.g., attention, appraisals) to later stages (e.g., memory, language, action tendencies, behaviors).
3. A facet approach should be prioritized over a total score approach to better distinguish the characteristics of alexithymia-related disturbances and to better specify treatments.
4. Alexithymia mainly involves a deficit in emotion processing, but emotion overresponding also occurs, with expression occurring either externally (i.e., crying, impulsive violence) or internally (i.e., avoidance strategies).
5. Most disturbances are observed when processing emotional stimuli, but there are also differences related to nonemotional material (e.g., memory, executive functions).
6. Although most outcomes are dysfunctional (i.e., maladaptive), there are cases in which responses are more functional (i.e., adaptive) in high alexithymia than in low alexithymia.
7. Although alexithymia is a stable personality trait, the fact that its stability is relative rather than absolute allows practitioners to develop interventions to reduce dysfunction and its societal impact. The treatment of primary alexithymia is in its infancy.
8. Interventions can take various forms, including psychotherapy, cognitive training, exercise, neurostimulation, and neurofeedback, with multi-modal approaches holding the most promise for efficacy.

## FUTURE ISSUES

1. There is a need to universally adopt a systematic, experimental approach to the study of alexithymia, including interdisciplinary studies utilizing designs with multiple processes, measures, and tasks.
2. The distinctions between what conditions and contexts result in deficits versus over-responding are important to further developing the alexithymia construct, particularly for clarifying functional over-responding as resilience to disease (rather than vulnerability), and potential therapeutic directions.
3. Alexithymia research needs better integration with affective and neurocognitive sciences.
4. Limited data show that alexithymia effects differ by context and time, indicating the urgent need to better characterize long-term outcomes.
5. Randomized controlled trials are needed to compare alexithymia-specific treatment approaches that target facet-level responses and behaviors and measure facet-level outcomes.
6. As contrasting effects often occur with positive, negative, and neutral stimuli, studies are needed that systematically consider these factors within the same sample and study design.
7. An important goal is to disentangle the influences of alexithymia relative to emotion state and context from the effects relative to stimulus and content valence.
8. Future study is needed of the interpersonal and collective influences of alexithymia (e.g., empathy, prosocial behaviors, communication skills).

## Abbreviations

Alexithymia: difficulty recognizing and/or describing one’s emotions; literally, “a lack of words for feelings” (*a*, no; *lexis*, language; *thymos*, emotion)
Pensée opératoire: operational thinking; a style of thinking that is utilitarian, pragmatic, abstract, factual, action oriented, and devoid of fantasy
Deficit: reduction or absence of emotional responding in contexts where such response is required
Facets (of alexithymia): the core domains of alexithymia, minimally including difficulty identifying feelings, difficulty describing feelings, and externally oriented thinking
Stability: the magnitude of score changes over time (absolute) versus the endurance of relative differences among individuals over time (relative)
Interoception: the sensation of one’s internal bodily signals and organs, such as heartbeat
Poor fantasy (PF) life: constricted imaginal processing often described in alexithymia, whose role as a distinct core facet of alexithymia is debated
Emotion regulation: implicit and explicit processes engaged to modulate one’s response to an emotional stimulus, which can be either adaptive or maladaptive
Attachment: emotional bonds between one person and another, established initially in infancy with one’s caregiver(s)
Appraisal: subjective evaluation of the personal significance of situations, objects, events, etc.
Executive functioning (EF): an umbrella term encompassing cognitive control processes for goal-directed behavior, including working memory updating, monitoring, task switching, and inhibitory control
Action tendencies: urges or readiness to carry out certain behaviors when confronted with emotion or threat
Experiential avoidance (EA): the tendency to avoid feelings, thoughts, sensations, and other internal experiences that might cause distress
Affect intensity: the magnitude of emotion expression when confronted with highly relevant appraisals, such as novel, unexpected, or important events
Affect frequency: the number of similar emotional occurrences or expressions in a particular period of time
Over-responding: excessive responding to emotion that can be maladaptive or adaptive depending on the context
Salience: the degree of prominence, importance, novelty, meaningfulness, or unexpectedness of a stimulus or event
Cognitive behavioral therapy (CBT): a psychotherapy approach directed toward discovering and changing maladaptive ways of thinking and behavior patterns through learning better coping strategies
Dialectical behavior therapy: structured psychotherapy focused on education, skill building, dialogue, and acceptance of the complexity of life and fluctuating feelings
Mindfulness: a psychotherapy component that focuses on awareness and acceptance of ongoing internal states, thoughts, feelings, and surroundings
